# Clinical Implications of Anemia Among Eastern Sudanese HIV Survivors

**DOI:** 10.7759/cureus.60766

**Published:** 2024-05-21

**Authors:** Bashir A Bashir

**Affiliations:** 1 Hematology, Port Sudan Ahlia University, Port Sudan, SDN

**Keywords:** sudan, port sudan, aids, anemia, hiv

## Abstract

Background

In HIV patients, anemia is the most common hematopoietic consequence, and it has a major influence on life satisfaction, morbidity, and survival. The epidemiology of anemia in this cohort in Sudan, however, is poorly characterized.

Aim

This study aims to assess the prevalence of anemia and its implications among HIV-positive patients.

Methods

An observational case-control study was administered in 44 clinical HIV-positive cases at the Sudan National AIDS Program (SNAP), Red Sea State, Sudan, between January 2018 and December 2019. A total of 44 HIV-negative subjects as controls were enrolled. HIV-infected patients’ demographic and clinical data, involving anemia and its outcome, were examined. WHO threshold was used to rank the clinical course of anemia in these patients.

Results

Of the 44 HIV patients examined, the mean age was 33.0 ± 11.2 years. Thirty (68.1%) were males, and 14 (29.8%) were females. The overall prevalence of anemia was 63.6% (95% CI 57.8-69.94%): mild grades of anemia at 25% (95% CI 14.4-34.4%), moderate grades of anemia at 36.4% (95% CI 28.7-43.2%), and severe anemia at 2.3% (95% CI 1.3-3.3%). In this study, 18 (64.3%) anemic patients showed normocytic thin smear pictures, among whom 44.3% were males and 20.5% were females. Microcytic anemia was seen in 17.8% of patients, while autoimmune hemolytic anemia was also detected in 17.8% of patients. The prevalence of anemia increased significantly with decreasing total lymphocyte count (TLC): 6.8% and 13.6% among patients with TLCs <1,000 and 1,000-4,800 cells/mm^3^, respectively (P = 0.016). Male sex was significantly associated with increased odds of anemia (OR 1.90, P = 0.049).

Conclusion

Anemia is a public comorbidity among HIV patients in the east of Sudan. Normocytosis is the most prominent form of anemia related to hypoproliferation in eastern Sudan, an event that can be triggered either by HIV chronicity or its combination with nutritional inadequacies. To prevent anemia progression and promote quality lifestyles, timely screening and appropriate therapy for anemia are critical, particularly for those with a low TLC.

## Introduction

Anemia is the most prominent hematopoietic anomaly associated with HIV disease, impacting 60-80% of patients with advanced clinical courses [1]. Whereas anemia may emerge as a simple laboratory aberration in some people, others would experience symptoms (such as fatigue, dyspnea, decreased exercise tolerance, and limited functional capacity) that are closely connected to a drop in hemoglobin concentration [2]. Impaired erythrocyte production, increased erythrocyte elimination, and inefficient erythrocyte generation may all play a critical role in the pathogenesis of HIV-associated anemia. Broadly speaking, these consist of infections or tumors invading the bone marrow, decreased endogenous erythropoietin production, hemolytic anemia, and using myelosuppressive medications such as zidovudine (ZDV) or as a result of taking numerous medications [3]. Anemia has been documented in HIV patients for a variety of reasons, the most prevalent of which being mineral, iron, and vitamin B12 deficiencies, as well as hookworm infestation, malaria infection, vitamin A insufficiency, genetic abnormalities, and comorbidity of TB and HIV [4]. In HIV-positive individuals, anemia lowers survival, accelerates the progression of the illness, and causes death (HR 2.6, 95% CI 1.9-3.4) [5]. Minimal studies have been conducted in developing nations to address this issue [3,6]. Anemia is independent of CD4+, T-lymphocyte count, and plasma HIV RNA concentration in the interaction between anemia and decreased survival. HIV-infected patients who overcome anemia have a higher chance of survival than those who do not [7]. African American ethnicity, age, overweight, history of pneumonia, oral candidiasis, history of fever, ZDV usage, low CD4+ cell counts (200 cells/mL), and greater HIV-1 RNA levels in plasma all enhance the risk of anemia. Hematological diseases, particularly anemia, have been implicated in ZDV usage, with the majority of cases arising within four to 12 weeks of starting the drug [8]. The focus of the research was to determine the presence of anemia and its consequences in HIV-positive individuals.

## Materials and methods

Study design and setting

This was a case-control study achieved at the Sudan National AIDS Program (SNAP), Red Sea State, Sudan, from January 2018 to December 2019. It is one of the state’s most known clinics, focusing on HIV treatment and extending health services to patients from all across the state. This facility not only offers health-related services to HIV-positive people but also inspires them to combat HIV and AIDS. It empowers HIV-positive individuals and communities to protect themselves, care for others, advocate for better services, and combat stigma and discrimination. A total of 44 HIV-positive patients have been recently confirmed, and 44 (control) subjects who are HIV-negative were studied. HIV-infected patients above the age of 15 were included in the study. We eliminated those with inadequate medical notes, pregnant women, acute systemic illnesses, blood transfusion patients, and patients who initiated antiretroviral treatment.

Data collection

Forms for standardizing data collection were filled out. Patient interviews yielded demographic and clinical information. Demographic information was acquired, including age, gender, marital status, education level, stage of HIV infection and AIDS according to the statement of WHO stage HIV disease [9], high-risk behaviors for HIV acquisition, and geographical area. Evaluations were conducted on the clinical information, including hypertension, diabetes, and opportunistic infections, as well as laboratory profiles, including hemoglobin (HB), hematocrit (HCT), mean corpuscular volume (MCV), mean corpuscular HB, mean corpuscular HB concentration, red cell distribution width coefficient of variation (RDW-CV), red blood cell (RBC) count, reticulocyte count, absolute reticulocyte count (ARC), corrected reticulocyte count (CRC), reticulocyte production index (RPI), and total lymphocyte count (TLC). For eligible anemic HIV, additional biomarkers of the iron profile and the serum levels of B12 and folic acid were undertaken.

Anemia evaluation and RPI

Anemia is a global public health issue that affects people of all ages in both developing and poorer nations. Anemia is defined by WHO as HB values of 12.0 g/dl in females and 13.0 g/dl in males. It was classified as mild 11-11.9 g/dl, moderate 8-10.9 g/dl, and severe 8 g/dl for males and 12.0 g/dl (mild 11-11.9 g/dl, moderate 8-10.9 g/dl, and severe 8 g/dl) for females [9]. Several biomarkers, primarily MCV and RDW-CV, were utilized as references to categorize the condition of anemia. The following formulae were adopted to define the CRC and RPI: CRC = reticulocyte count (patient’s HRT or normal HRT) and RPI = CRC/correction factor. The maturation correction factor was 1 for HCT of 36-45%, 1.5 for HCT of 26-35%, 2 for HCT of 16-25%, and 2.5 for HCT <15% [10].

Laboratory method

Three milliliters of venous blood were collected aseptically from every patient and treated with di-potassium ethylene diamine tetra-acetic acid (K2EDTA) for complete hemogram determination using a three-part semiautomated hematology analyzer (Sysmex KX 21N, Japan).

Statistical analysis

Mean ± SD is being used to express continuous data. In each category, categorical variables were reported as numbers and percentages. Anemia prevalence was expressed as a percentage with a 95% CI. Fisher’s exact and χ2 tests assessed the significance of differences between categorical variables, while t-tests were used for continuous data. To compare RPIs pairwise within the anemic or nonanemic group at different points, paired t-tests were employed. The OR of 95% CI was used to determine sex association with anemia among people with positive HIV. All statistical analysis was performed with IBM SPSS Statistics for Windows, Version 25.0 (Released 2017; IBM Corp., Armonk, NY, USA). Statistical significance was defined as p < 0.05 for all two-tailed tests.

Ethical approval

The current study fulfilled the Helsinki Declaration and was issued by the Ministry of Health (letter number 44/T/X7) and the SNAP in the Red Sea State, Sudan. All HIV-infected patients signed an informed consent form.

## Results

This study recruited 44 HIV-positive patients. The mean (SD) age was 33 ± 11.1 years, with males accounting for 30 (68.0%) and females accounting for 14 (29.8%). The control group’s mean (SD) age was 28.7 ± 10.8 years, with males being 32 (72.7%) and females representing 12 (27.3%). Table 1 summarizes the baseline features of the analyzed findings. The Hadandawa tribe has the highest rate of HIV infection (59%), followed by the Bani Amer tribe (20.5%) and the western Sudan tribe (15.9%). The Hadandawa tribe has the greatest prevalence of anemia related to HIV infection (40.9%).

**Table 1:** Comparison of anemic and nonanemic HIV/AIDS patients’ demographic and laboratory data The data has been represented as N, %, and mean ± SD; a p-value is considered significant (p < 0.05). HB, hemoglobin; HCT, hematocrit; MCH, mean corpuscular hemoglobin; MCHC, mean corpuscular hemoglobin concentration; MCV, mean corpuscular volume; RBC, red blood cell; RDW-CV, red cell distribution width coefficient of variation; TIBC, total iron binding capacity; TLC, total lymphocyte count; UIBC, unsaturated iron binding capacity

Characteristics	Anemic	Nonanemic	Control	P-value
	(n = 28)	(n = 16)	(n = 44)	
Sex				
Male	16 (36.4%)	14 (31.8%)	32 (72.7%)	0.0001
Female	12 (27.3%)	2 (4.5%)	12 (27.3%)	
Age (mean ± SD)	33.5 ± 11.7	32.2 ± 10.3	28.7 ± 10.7	0.002
Marital status				
Single	13 (46.4%)	9 (56.2%)	19 (43.2%)	0.377
Married	15 (53.8%)	7 (43.8%)	25 (56.8%)	
Education				
Illiterate/primary	12 (42.9%)	5 (31.3%)	8 (18.2%)	0.647
High school/university	16 (57.1%)	11 (68.7%)	36 (82.8%)	
AIDS				
Yes	10 (35.7%)	1 (6.3%)	-	0.061
No	18 (64.3%)	15 (93.7%)	-	
Tribe				
Hadandawa	18 (40.9%)	8 (18.2%)	12 (27.3%)	0.097
Bani Amer	4 (9.1%)	5 (11.2%)	11 (25.0%)	
Northern tribe	0	2 (4.5%)	16 (36.4%)	
Western tribe	6 (13.6%)	1 (2.3%)	5 (11.3%)	
HB, g/dl	10.3 ± 1.3	13.1 ± 1.0	14.1 ± 0.89	0.0001
RBCs, µl	3.85 ± 0.56	4.53 ± 0.52	4.74 ± 0.60	0.0001
MCV, fl	78.8 ± 9.5	81.2 ± 6.4	90.3 ± 10.6	0.0001
MCV (morphologic)				
Normocytic	12 (42.8%)	13 (81.2%)	37 (84%)	0.0001
Microcytic	5 (17.9%)	2 (12.5%)	2 (4.6%)	
Dimorphic	5 (17.9%)	1 (6.3%)	2 (4.6%)	
Macrocytic	-	-	3 (6.8%)	
Schistocytic	6 (21.4%)	-	-	
MCH, pg	27.9 ± 4.8	30.0 ± 3.2	30.3 ± 3.5	0.057
MCHC, %	35.0 ± 2.2	36.9 ± 1.8	33.6 ± 3.8	0.003
HCT, %	29.5±3.2	36.5 ± 3.0	42.7 ± 5.6	0.0001
RDW-CV, %	16.7 ± 3.3	14.9 ± 2.3	14.0 ± 1.6	0.0001
TLC, µl	1,469 ± 842	2,000 ± 808	4,806 ± 1,397	0.0001
Reticulocyte index				
Reticulocyte count, %	0.38 ± 0.08	0.5 ± 0.62	0.5 ± 0.71	0.105
Absolute retic count, µl	14.6 ± 5.7	22.7 ± 8.9	26.0 ± 10.2	0.021
Corrected retic count, %	0.3 ± 0.07	0.4 ± 0.09	0.53 ± 0.12	0.09
Retic production index	0.2 ± 0.07	0.3 ± 0.11	0.5 ± 0.18	0.077
Iron profile				
Iron, µg/dl	86.7 ± 43	78 ± 39	91 ± 22	0.096
Ferritin, µg/dl	89 ± 49	82 ± 40	114 ± 13	0.082
TIBC, µg/dl	309 ± 67	289 ± 53	313 ± 84	0.135
UIBC, µg/dl	220 ± 50	211 ± 14	222 ± 62	0.098
Transferrin saturation, %	26.8 ± 14	27 ± 12	32 ± 12	0.053
Vitamin B_12_	431 ± 118	388 ± 108	517 ± 110	0.211
Folic acid	17.8 ± 6.2	14.7 ± 3.6	15.3 ± 4.1	0.467

The overall prevalence of anemia was 63.6% (95% CI 57.8-69.94%), with mild anemia accounting for 25% (95% CI 14.4-34.4%), moderate anemia accounting for 36.4% (95% CI 28.7-43.2%), and severe anemia accounting for 2.3% (95% CI 1.3-3.3%) (Table 2). The vast majority of patients experience mild to moderate anemia. Mild to moderate anemia struck 25.0% and 36.4%, respectively.

**Table 2:** Degree of anemia The data has been represented as N, %, and 95% CI.

Grade	Frequency	Prevalence, %	95% CI	
		95% CI	Lower limit	Upper limit
Anemia	28	63.6	57.8	69.9
Anemia intensity (n = 28)				
Mild	11	25	14.4	34.4
Moderate	16	36.4	28.7	43.2
Severe	1	2.2	1.3	3.3

The average age in this study was 33.0 ± 11.2 years. The categorical analysis found no statistically significant trend between anemia and age (P = 0.532). On the other hand, our study found a substantial link between sex and anemia, with males having a considerably higher risk of getting anemia (OR 1.90, P = 0.049).

**Figure 1:**
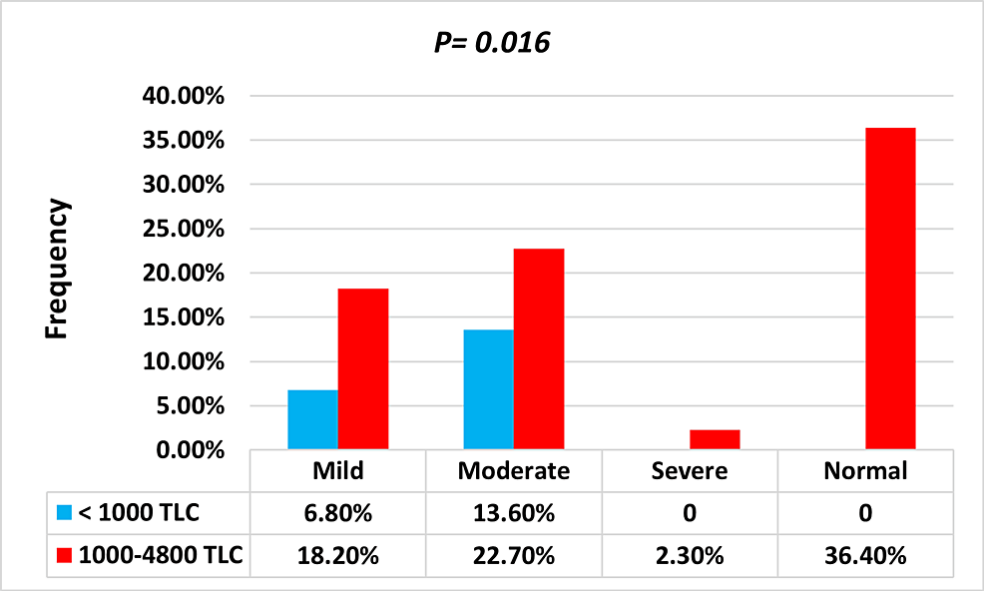
Link between anemic intensity and TLC (χ2) TLC, total lymphocyte count

The patients’ HB levels varied between 5.8 and 15.2 g/dl. In anemic and nonanemic patients, HB’s mean (SD) was 10.3 ± 1.3 and 13.1 ± 1.0 g/dl, respectively. The difference, however, was statistically significant (P = 0.0001). In this study, 18/28 (64.8%) anemic patients were normocytic (male = 44.3%, female = 20.5%). Microcytic anemia was present in 17.8%, while autoimmune hemolytic anemia was also found in 17.8% (Table 1). Females were more likely than males to have microcytosis (20.5% vs. 11%; P = 0.008). The mean (SD) TLC for HIV anemic cases was 1,469 ± 842 cells/mm^3^, while it was 2,000 ± 808 cells/mm^3^ for nonanemic HIV cases. In this experiment, all HIV cases exhibited significantly decreased TLC as compared to controls. Also, the mean TLC in anemic patients was dramatically lower than in nonanemic patients. Anemia prevalence increased with lower TLC (as immunostatus) among patients with TLC 1,000 and 1,000-4,800 cells/mm^3^, and the difference was statistically significant (P = 0.016) (Figure 1). We found a substantial correlation between anemia and TLC (P = 0.048) as well as individual gender (P = 0.038). Table 1 and Table 2 incorporate additional information.

Among all anemic individuals, 18% exhibited ferritin deficiency (reference value 20-250 ng/dl), and 3.6% had vitamin B12 deficiency (reference value 160-950 pg/ml). There was no patient with a folic acid deficit (reference value 2.7-17 ng/mL). The mean ± SD values for serum iron, ferritin, total iron binding capacity, transferrin saturation, folic acid, and vitamin B12 in anemic patients were 86.7 ± 43.0 µg/dl, 89 ± 49 ng/dl, 309 ± 67 µg/dl, 26.8 ± 14%, 17.8 ± 6.2 ng/ml, and 431 ± 118 pg/ml, respectively.

Additionally, compared to nonanemic HIV, the reticulocyte count, ARC, CRC, and RPI of HIV patients were considerably lower (P = 0.077) at 0.38 ± 0.08%, 14.6 ± 5.7 × 103/µl, 0.3 ± 0.07%, and 0.2 ± 0.07%, respectively.

Out of 44 HIV-positive patients that were recruited, 10 (22.7%) were in a late HIV stage (AIDS); the male-to-female ratio was 7 to 3. AIDS patients frequently had anemia (10/28; 35.7%); however, this difference was not statistically meaningful (P = 0.061). More knowledge has also been displayed in Table 1.

## Discussion

WHO assessment and projections for 2020 set the HIV epidemic in Sudan as a minimal epidemic, with an adult (15-49 years) HIV prevalence of about 0.2% [11]. Anemia is an emerging consequence of HIV-1 infection that could have clinical implications for public health. Anemia etiology is multifaceted, making it difficult to pinpoint the underlying cause and thus limiting treatment [12]. Anemia patterns vary by study setting but have been reported to be as high as 85% [4]. The overall prevalence of anemia in this study was 63.6%, which is consistent with previous studies from various spots that found high rates: 64% in Nigeria [13], 71% in Isfahan, Iran [4], 69.7% in Benin [14], and 77.4% in Tanzania [15]. Even so, our percentage is higher than that of a number of other Asian and non-Asian countries, such as China (51.9%) [16], Ethiopia (34.6%) [17], northern India (16.2%) [18], South Africa (25.8%) [8], and Ghana (23.8%) [19].

Our results showed that mild, moderate, or severe anemia affected 25%, 36.4%, and 2.2% of the patients, respectively. The prevalence of mild anemia (25%) found in this study does not match the findings of another study (14%) [4]. However, this proportion was below the findings of a Chinese study (32.4%) [16]. A total of 36.4% of the anemic participants in this study had moderate anemia. In previous research, lower rates of moderate anemia were witnessed: 17% in China [16] and 15.6% in Ethiopia [17]. This study’s rate of severe anemia (2.2%) is lower than prior research, which found 2.55% in China [16], 5% in Ethiopia [17], and 4% in Iranian HIV-infected patients [4]. Moreover, a decline in the consistency of observed prevalence may have resulted from lower HB concentration criteria used in earlier studies to identify anemia. Anemia was classified in several of these studies as HB 12 g/dl for males and 11 g/dl for females [16]. As a result, the lower thresholds may have significantly reduced the prevalence of anemia while underestimating the extent of the problem.

Peripheral blood smears have been investigated since they contain a wealth of information on cell morphology and can provide insights into the etiology of the hematopoiesis alteration. According to our findings, the much more prominent type of anemia in HIV-positive cases in eastern Sudan is normocytic anemia. Medication toxicity, viral suppression, malignant marrow infiltration, combined nutritional deficiencies, and chronic infections can all induce this type of anemia. According to Mata-Marin et al., the most common kind of anemia (85%) in HIV-positive patients is normocytic normochromic anemia, and there was no evidence of macrocytic anemia in their study [20]. These findings were considerably consistent with our outcomes.

The average age in this study was 33.0 ± 11.2 years, which was consistent with past research [18]. The categorical analysis found no statistically significant trend between anemia and age (P = 0.532). A prior study’s findings also corroborate this nonsignificant connection [9]. There are no definitive implications about the link between sex and anemia in HIV infection. Past research made no distinction between sex and anemia; nonetheless, the prevalence of anemia was higher in females than in males [21]. On the contrary, our study found a substantial link between sex and anemia, with males having a considerably higher risk of getting anemia (OR 1.90, P = 0.049). Furthermore, this connection is relatively confirmed by previous findings in other study contexts [22]. The increased rate of anemia and its elevated chance in HIV-seropositive patients may be attributed to the fact that females of fertile age are more likely to experience blood loss from menstruation and increased blood supply demands, resulting in an elevated rate of anemia.

TLCs in our cases were found to be considerably decreased in anemic patients compared to nonanemic patients (1,469 ± 842 versus 2,000 ± 808, P = 0.0001). Categorical analysis, on the other hand, revealed a significant relationship between TLC and anemia, indicating an immunostatus (P = 0.016). Our outcomes, however, were consistent with those of a previous study [22]. The intensity of anemia was highly connected with TLC in this study, and the proportion of onset anemia rose with decreasing TLC. This could be an important clinical implication of our findings, in which reduced TLC was linked to an increased likelihood of anemic intensity.

Furthermore, our research found that, similarly to Oluboyo et al. [23], there was no discernible variation in iron markers among HIV cases. Not too long ago, the bone marrow released immature RBCs called reticulocytes. To determine the efficiency of erythropoiesis and the productivity of bone marrow, the reticulocyte count should be adjusted to produce an RPI. RPI is a useful metric for evaluating erythropoiesis. An RPI >2 denotes robust erythropoiesis, whereas an RPI <2 denotes minimal erythropoiesis (hypoproliferation) [11]. In the current study, a significantly lower RPI was noted in those who developed anemia as compared to those who did not develop anemia. Nonetheless, our findings agreed with those of an earlier investigation [24].

In HIV patients, the conclusion is that anemia is a common concern. HIV infection tends to be related to hypoproliferative anemia, which is characterized by normochromic, normocytic anemia with a low reticulocyte count and a normal iron reserve. HIV can directly decrease the endogenous erythropoietin response and produce inflammatory cytokines in the bone marrow, which in turn depress hematopoietic progenitor cells. Thus, anemia is indeed a relevant factor when considering prognostic indices for mortality and morbidity in HIV patients. Impacting morbidity, anemia can lead to reduced quality of life and functional impairment. It may exacerbate other health conditions and affect overall well-being. Impacting mortality, researchers have shown a correlation between anemia and increased mortality risk among HIV survivors.

The relatively small sample of HIV patients, the neglect of pathological changes, the quantification of HIV viral load, and the CD4/CD8 count are all limitations of this study.

## Conclusions

Among HIV patients in Sudan, anemia is a common comorbidity. The prevalence of anemia in this study is higher than in other studies of a comparable nature. In eastern Sudan, normocytosis is the most common type of anemia associated with hypoproliferation. Nutritional deficiencies combined with HIV chronicity may be the cause. Appropriate treatment and diagnosis of anemia are essential for stopping its progression and promoting healthy lifestyles, especially in individuals with low TLCs.
